# Assessing the long-term effects of microbial therapeutics as treatment within psychiatry: a systematic review

**DOI:** 10.3389/fpsyt.2025.1663719

**Published:** 2026-01-12

**Authors:** Cassandra Sgarbossa, Arthi Chinna Meyyappan, Evan Forth, Hayley Bromley, Roumen Milev

**Affiliations:** 1 Centre for Neuroscience Studies, Queen’s University, Kingston, ON, Canada; 2 Providence Care Hospital, Kingston, ON, Canada; 3 Department of Psychiatry, Queen’s University, Kingston, ON, Canada; 4 Department of Psychology, Queen’s University, Kingston, ON, Canada

**Keywords:** microbial therapeutics, novel treatments, longitudinal effects, mood disorders, quality of life, psychiatric symptoms, fecal microbiota transplantation, probiotics

## Abstract

**Background:**

The management and treatment of psychiatric disorders by manipulating the gut microbiome and utilizing microbial therapeutics, via modulation of the gut-brain-axis, has been a rapidly growing field of research. Given the novelty of using microbial therapeutics within psychiatry, a growing number of studies have investigated their use as treatment for various psychiatric disorders and symptoms. However, few studies have explored the longitudinal efficacy of these treatments. This review aims to summarize the findings of any studies assessing the long-term effects of gut-related interventions on mood and psychiatric symptoms.

**Methods:**

A systematic search of 4 databases (Embase, PsycINFO, Medline, Web of Science) from inception to May 28, 2025, informed by Preferred Reporting Items for Systematic Reviews and Meta-Analyses (PRISMA) guidelines, and using key words relating to microbial therapeutics, psychiatric disorders, and long-term effects was conducted. Findings were included or excluded using pre-determined eligibility criteria such as being been written in English and published by a peer-reviewed journal, assessed for quality using the Cochrane Handbook for Systematic Reviews of Interventions Risk of Bias tool, and qualitatively evaluated.

**Results:**

The search yielded 4175 studies, of which 1274 duplicates were removed. All remaining studies underwent abstract screening, from which 70 records were full-text screened and a total of ten clinical studies (*n* = 10) met eligibility criteria and were included in the review. The majority of studies explored the effects of microbial therapeutics such as fecal microbiota transplant and probiotics, as treatment for disorders of the gastrointestinal tract as the primary scope, with additional outcome measures assessing psychiatric well-being. The review presented with mixed findings: many studies reported a sustained improvement in symptoms of depression and anxiety ranging from 3- to 18-months post-treatment, while others reported the opposite with no sustained long-term improvement in mood-related symptoms. There was also a lack of consistency across follow-up duration between studies, making it difficult to compare findings.

**Conclusions:**

Overall, this review highlighted the need for more placebo-controlled studies with larger sample sizes to effectively evaluate the longitudinal potential of microbial therapeutics as treatment for mood-disturbances and psychiatric symptoms. With consideration for the limitations of this field, these results provide evidence that there may be long-term benefits of targeting the gut microbiome as treatment for mood-related disturbances.

## 1 Introduction

Globally, approximately 1 in 8 individuals lived with a psychiatric disorder before the COVID-19 pandemic (1). This involves a variety of psychiatric disorders ranging anywhere from eating disorders, to neurodevelopmental disorders, or even mood disorders such as Major Depressive Disorder (MDD), Bipolar Disorder, or Seasonal Affective Disorder (SAD) to name a few. During the onset of the pandemic, there was an estimated global addition of 53.2 million cases of MDD, with an increased proportion of female cases at 29.8% than male at 24%, and an overall increase of 27.6% of MDD cases worldwide (2, 3). Considering these staggering statistics, it is without surprise that the relative economic burden and total output loss of psychiatric disorders are projected to cost $16.3 trillion USD between 2011 and 2030, worldwide (4). The prevalence of mood disorders and their impact on society highlights the need for a better understanding of psychiatric disorder pathology, as well as the need for more accessible treatment options and resources for individuals with a psychiatric disorder. Currently, there are a variety of conventional treatments available to alleviate symptoms of psychiatric disorders. These include but are not limited to cognitive behavioral therapy (CBT), pharmacological treatments such as antidepressant medications, and neurostimulation treatments such as electroconvulsive therapy (ECT), repetitive transcranial magnetic stimulation (rTMS), or vagus nerve stimulation (5, 6). Unconventional treatment methods such as microbial therapeutics, which modulate the gut microbiome through microbiota manipulation, have only recently gained attention in the psychiatric field for their influence on symptoms of psychiatric disorders and their potential to provide a more personalized and integrated approach to treatment (7).

The human gut is comprised of over 100 trillion commensal bacteria, which coexist symbiotically throughout the body and central nervous system (8). While microbiota refers to the bacterial species living in a community, the gut microbiome is an umbrella term that includes not only the microbiota, but their structural and genetic elements, metabolites, and the greater environment in which they interplay with (9). The gut microbiome is constantly in communication with, and giving feedback to, various pathways and systems throughout our body such as the autonomic nervous system, enteric nervous system, immune system, and other communities of microbes and distinct microbiomes (10, 11). While the majority of the gut is comprised of two dominant phyla of bacteria (10, 12), Firmicutes and Bacteroides, the microbiota within the human intestine remains incredibly diverse to include many other types of bacteria such as Proteobacteria, Actinobacteria, Fusobacteria, and Verrucomicrobia (10). Furthermore, the communication between the gut microbiome and the brain that is referred to as the ‘gut-brain axis’ (GBA), exists as a bidirectional signaling pathway between the gastrointestinal tract (GI) and the brain (7).

As a result of communication through the GBA, the gut microbiome plays a key role in maintaining an organism’s natural homeostatic balance (13). Given that microbiota are continuously providing feedback to the brain and interacting with our environment, certain environmental factors such as diet, stress, metabolism, geography, and genetics can both directly and indirectly influence our microbiome composition and microbial balance (7, 10, 13). While the exact mechanism by which these external factors can influence the GBA has yet to be fully elucidated, current research suggests that these effects are exerted through interactions with the immune, endocrine, metabolic, and nervous systems (10, 14–16). One example of the gut microbiome interacting with and influencing these various systems is through the production of short chain fatty acids (SCFAs). SCFAs are a metabolite produced by microbiota in the gut through the fermentation of non-digestible carbohydrates. SCFAs have been found to have anti-inflammatory properties and play a role in the regulation of many cells of the innate and adaptive immune system, including neutrophils, macrophages, T-cells and B-cells (17). They also influence the production and bioavailability of peripheral serotonin through interactions with enterochromaffin cells (18), and directly innervate neurons of the enteric nervous system, which affects key properties of the central nervous system such as vagal tone (19, 20). This is just one potential way by which the gut microbiome can influence a variety of systems in the body, resulting in an influence on overall health and microbial balance.

When there is an imbalance, or what is referred to as microbial dysbiosis, either due to environmental influences, toxins, antibiotic use, and/or pathogens, the protective epithelial gut barrier can become compromised (7, 21, 22). While a consensus has yet to be reached for the full characterization of what constitutes ‘eubiosis’ or a ‘healthy’ microbiome, ‘dysbiosis’ is still frequently used to describe a change in the gut microbiome that presents dissimilarly and/or with a microbial imbalance (23). Perturbations of this barrier can subsequently result in ‘*leaky gut*’ syndrome, where microbial imbalances increase the permeability of intestinal mucosa in the GI tract and allow possible bacterial toxins, toxic digestive metabolites, and small molecules to leak into the bloodstream (21, 24). This bacterial translocation has been shown to contribute to overall inflammation of the immune system from both acute inflammatory responses and sub-chronic inflammation (25–27). Leaky gut syndrome has also been associated with a number of health-related conditions, such as: autoimmune disorders, inflammatory bowel disease, diabetes, more recently psychiatric disorders such as depression, anxiety, ADHD, autism spectrum disorder (ASD), and other physical symptoms such as bloating, cramps, and fatigue (7, 21, 28–30). While microbial imbalances may raise concern for their widespread capability throughout the human body, they also serve as a steppingstone for recognizing microbial therapeutics as a possible treatment method for such conditions and symptoms.

When targeting the gut microbiome, there are both additive and subtractive treatment options in terms of repopulation parameters (31). Subtractive methods such as antibiotics, which are one of the most well-established methods of microbiota modulation, have long been used for addressing bacterial infections. However, given that antibiotics work by inhibiting and/or killing bacterial growth, they also carry the risk of disrupting our natural microbial balance and perturbing established communities of microbes, which may lead to gut dysbiosis (11, 32). On the contrary, additive microbial therapeutics aim to do the opposite and rather, encourage pre-existing bacterial growth, maintain general gastrointestinal health, and/or repopulate the gut with bacteria. Some of these techniques include but are not limited to prebiotics, probiotics, postbiotics, bacteriotherapy, and fecal microbiota transplantation (FMT) (31). Prebiotics aim to improve the growth and activity of bacteria in the body, whereas probiotic administration is the ingestion of live microorganisms which are expected to confer a health benefit to the host (33), potentially through interactions with the host’s microbiome. Postbiotics refers to the use of inanimate microorganisms and/or their components to confer a health benefit to the host (34), and bacteriotherapy is a general term referring to the use of bacterial measures as a treatment strategy for illnesses. Finally, FMT involves the transfer of fecal material from a donor to the intestinal tract of a recipient via colonoscopy or nasogastric tube (11, 35).

FMT has long been used as treatment for various GI-related medical conditions, such as C. Difficile infections, Irritable Bowel Syndrome (IBS), Ulcerative Colitis (UC), and Crohn’s Disease (CD) (35), however it is only recently that such gut-repopulation techniques have been identified as possible treatment options for psychiatric disorders. Furthermore, the existing commonality and co-occurrence of psychiatric-based disorders and other health-related conditions of the body, suggests a potential biological relationship between the two, potentially via the GBA. Past research has highlighted that depression and/or anxiety can both co-occur with IBS approximately 44-84% of the time (36), while other literature has documented the highly linked relationship between ASD’s and functional GI disruptions (37). Given the multitude of interactions between the GBA and other bodily systems, microbial imbalances could exert an outsized influence and significant impact on numerous peripheral and psychiatric symptoms.

Deriving from the monoaminergic hypothesis of depression, many conventional treatments such as antidepressant medications, are used to increase the bioavailability of monoamines in the central nervous system by either blocking the reuptake of monoamines in the synapse, inhibiting the metabolism and/or degradation of monoamines, or blocking the presynaptic inhibitory receptors (38). However, these treatments are often accompanied with a high rate of relapse and disorder reoccurrence following discontinuation of treatment (39). Baldessarini and Tondo (2019) reported that the immediate discontinuation of antidepressant medication corresponded to an average relapse time of 3 months post-discontinuation of treatment. However, when antidepressant treatment was gradually discontinued, the average rate of relapse was prolonged to 6-months post-discontinuation (39). ECT, which is used for its effective and rapid therapeutic outcome, still carry their own caveats as it too has a reported relapse rate of approximately 6 months (40). Cohen and colleagues (2009) found rTMS to yield an attractive 75% remission rate at 2-months post-treatment, however this rate dropped to 60% at 3-months post-treatment with only 22% of patients maintaining remission at 6-months post-treatment (41). While it may be likely for individuals to relapse following a discontinuation of treatment, comparing relapse rates across various treatment options is still difficult, as different treatments have varying frequencies of application and carry their own set of side effects that can also influence treatment adherence.

It is prominent however, that many available treatment methods for psychiatric disorders demonstrate a common relapse rate between the 3- to 6-month post-treatment timepoint. As idealistic as it may seem to find an ‘enchanting’ treatment method that could produce profound and lifelong rates of remission, it is neither practical nor realistic. A critical component to optimal care of many psychiatric disorders is prevention and long-term management. However, the innovation of utilizing a more holistic treatment approach such as microbial therapeutics, which use the body’s own bacterial components to mediate mood, certainly begs the question of their own relapse rates. As with most treatments, microbial therapeutics are not without their own caveats concerning treatment adherence and relapse rates. Literature has reflected that short-term adverse events such as bloating, flatulence, diarrhea, irritable bowels, constipation, abdominal pain, fever, and nausea can result from microbial therapeutics, which may impact an individual’s adherence to treatment (42–44). Nevertheless, identifying potential relapse rates for gut-related treatments is warranted for further understanding of their long-term impact. To assess this dynamic relationship, we have conducted a systematic review on the long-term effects of microbial therapeutics as treatment for symptoms of psychiatric disorders. Determining the longitudinal efficacy of microbiome-targeted treatments would provide valuable insight and be pivotal for evaluating gut-repopulation techniques as an option for psychiatric disorders. Though many studies have assessed the short-term effects of microbial therapeutics on psychiatric symptoms, a review of the literature focusing only the long-term psychiatric effects of these interventions is necessary to determine the longitudinal efficacy, identify potential long-term risks, and inform future clinical applications of such treatments.

## 2 Methods

### 2.1 Literature search strategy

This systematic review was informed by Preferred Reporting Items for Systematic Reviews and Meta-Analyses (PRISMA) guidelines and used Covidence, a primary screening and data extraction tool, to extract and screen data (45). Databases MedLine, PsycInfo, Embase, and Web of Science were searched using the following keywords: (depression OR depress* OR MDD OR bipolar* OR bipolar disorder OR ADHD* OR attention deficit hyperactivity disorder OR autism* OR ASD OR anxiety* OR GAD OR mania OR schizo* OR obsessive compulsive disorder OR OCD OR posttraumatic stress disorder OR PTSD) AND (probiotic* OR prebiotic* OR psychobiotics* OR fecal microbiota transplant* OR FMT OR stool transplant* OR bacteriotherapy OR microbe therapy OR microbe transfer OR microbial therap*) AND (follow-up OR long-term OR longitudinal*). The search was conducted from inception up to May 28^th^, 2025.

### 2.2 Eligibility criteria

Eligibility criteria for article inclusion is as follows (1): Articles must be written in English and published by a peer-reviewed journal (2); Population: Subjects in the study must be assessed for symptoms of a psychiatric disorder (3); Intervention: Subjects must have undergone a form of microbe transfer and/or received a microbial therapeutic product; (4) Outcome: Subjects must have a follow-up visit that includes clinical assessment conducted at a time point of 12-weeks or longer; (5) Study Design: Restricted to any type of clinical (e.g., RCT) and pre-clinical study. Exclusion criteria was any study that does not meet inclusion criteria.

#### 2.2.1 Study screening

Author C.S. completed all literature searches. Both authors C.S. and A.C.M completed the abstract and full-text screening independently based on eligibility criteria. Any conflicts between reviewers for full-text screening was resolved by a third party, R.M.

#### 2.2.2 Study quality

Quality assessment was conducted using the Cochrane Handbook for Systematic Reviews of Interventions, Risk of Bias tool. The Cochrane Risk of Bias tool assesses the risk of bias for 6 distinct domains: sequence generation, allocation concealment, blinding of participants and personnel, blinding of outcome assessment, incomplete outcome data, and ‘other’ sources of bias. Authors C.S. and A.C.M. completed the quality assessment for all studies. The majority of studies presented with an overall high risk of bias in the sequence generation, allocation concealment, and blinding domains. This was anticipated due to the scope of the systematic review, as there is a limited body of literature examining microbial therapeutics as treatment within a psychiatric setting. For these reasons, case studies were not excluded from the review, in efforts to retain as much data as possible on any longitudinal effects. These case studies also inherently increase the overall level of bias for the review, as there is no blinding or sequence generation in case studies.

#### 2.2.3 Data extraction & analysis

All included studies were independently assessed by two investigators through full text.

screening. The following information was obtained and extracted: year and author information, population information (sample size, primary illness indication), study design characteristics (study design, intervention, follow-up timepoints), and clinical scale assessments (psychiatric measures and questionnaires). Data was analyzed using a qualitative approach, due to the quantity of studies and in efforts to gain supplementary information in a novel and heterogenous subject area.

## 3 Results

### 3.1 Study selection

**Figure 1:**
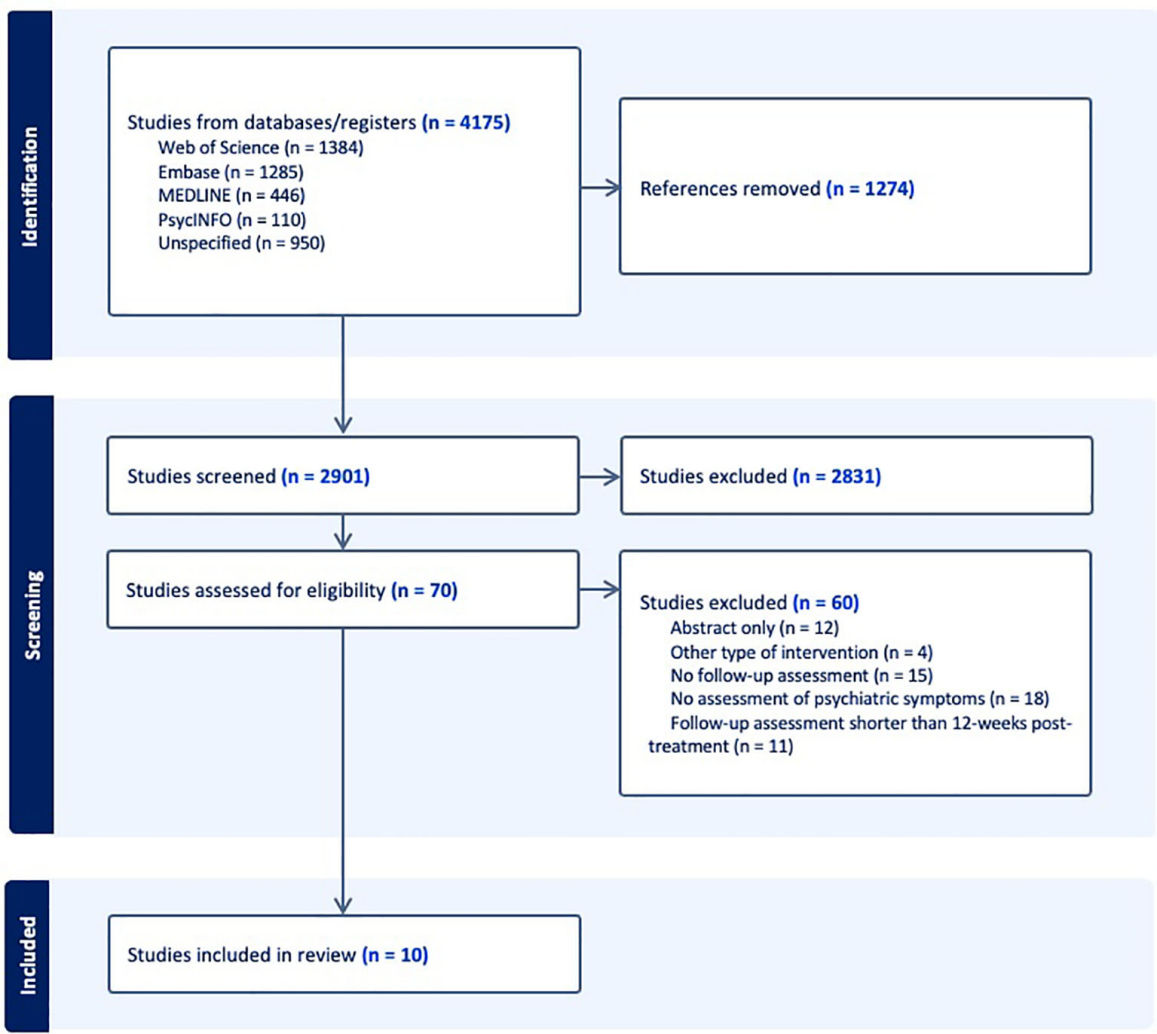
Detailed description of PRISMA flow diagram.

The initial literature search yielded 4175 studies, of which 1274 duplicates were removed, resulting in 2901 studies to be screened. In total, 70 studies passed abstract screening and underwent full-text review, from which 60 were excluded for either: no assessment of psychiatric symptoms, no follow-up assessment, abstract only, follow-up assessment less than 12 weeks post-treatment, or other type of intervention. This review will include the details of the remaining 10 articles that met eligibility criteria. Please refer to **Figure 1** for a detailed description of the above PRISMA screening process. The 10 eligible studies were assessed for quality using the Risk of Bias tool. A detailed summary of the quality assessment can be found in **Table 1**.

**Table 1:** Detailed summary of quality assessment characteristics.

Study	Sequence generation		Allocation concealment		Blinding of participants and personnel		Blinding of outcome assessment		Incomplete outcome data		Selective reporting	
	ROB	Comment	ROB	Comment	ROB	Comment	ROB	Comment	ROB	Comment	ROB	Comment
Lin (2021) (51)	N/A	No mention of how sequence was generated.	N/A	No mention of how allocation was concealed.	High	No description of personnel blinding, however participants appeared to remain blinded.	N/A	No mention of if outcome assessors were blinded.	Low	Excluded subjects were documented with reasoning.	Low	N/A
Huang (2022) (54)	High	Single case study - N/A	High	Single case study - N/A	High	No mention of blinding.	High	No mention of blinding.	Low	Single case study, no exclusions reported.	Low	N/A
Mizuno (2017) (49)	High	Open-label, single arm study.	High	Open-label, single arm study. No allocation concealment.	High	No blinding to participants or personnel.	Low	Outcome assessor was blinded.	Low	Excluded subjects were documented with reasoning.	Low	N/A
Lahtinen (2020) (46)	Low	Participants randomized 1:1 in blocks of six.	N/A	No mention of how allocation was concealed.	Low	Both personnel and participants were blinded.	N/A	No mention of who performed the outcome assessment.	Low	Excluded subjects were documented with reasoning.	Low	N/A
Huang (2019) (47)	High	Single arm design, no sequence generation.	High	Single arm design, no allocation concealment.	High	No mention of blinding.	High	No mention of blinding.	Low	No reports of excluded and/or withdrawn participants.	Low	N/A
Johnsen (48) (2020)	Low	Participants randomized in blocks of six for active or placebo (4:2). Non-study personnel generated randomization via website.	Low	Participants, investigators, and outcome assessors were kept blind to the allocation and intervention.	Low	Participants, investigators, and outcome assessors were kept blind to the allocation and intervention.	Low	Participants, investigators, and outcome assessors were kept blind to the allocation and intervention.	Low	Excluded subjects were documented with reasoning.	Low	N/A
Xie (2019) (50)	High	Single case study - N/A	High	Single case study - N/A	High	No mention of blinding.	High	No mention of blinding.	Low	Single case study, no exclusions reported.	High	Results were mentioned but lack of statistics. This was due to the nature of the article being a single case study.
Cai (2019) (53)	High	Single case study - N/A	High	Single case study - N/A	High	No mention of blinding.	High	No mention of blinding.	Low	Single case study, no exclusions reported.	High	Results were mentioned but lack of statistics. This was due to the nature of the article being a 'letter to editor' single case study.
Ghorbani (2022) (52)	Low	Non-study personnel prepared randomized blocks of 4, using the sas proc plan algorithm.	Low	Participants, coordinators, and PI's were blinded.	Low	Participants, coordinators, and PI's were blinded.	N/A	No mention of if outcome assessors were blinded.	Low	Excluded subjects were documented with reasoning.	Low	N/A
Wang (2023) (53)	High	No mention of sequence generation.	High	No mention of allocation concealment.	High	No mention of blinding.	High	No mention of blinding.	High	No mention of subjects that were excluded	High	No mention of excluded subjects or their results that may have not been included.

### 3.2 Study characteristics

The characteristics and detailed summary of each included study is displayed in **Table 2**. A total of ten clinical studies with human samples were included in this review, as there were no pre-clinical studies that matched eligibility criteria. The majority of studies in this review primarily explored the effects of microbial therapeutics for GI-related health conditions, with additional outcome measures assessing psychiatric well-being. This included any population that has either assessed for psychiatric disorders (e.g., ASD) or severity of psychiatric manifestations (e.g., symptoms of anxiety). Given the novelty of the field, few studies have assessed the use of microbial therapeutics within a psychiatric setting, let alone examining the associated long-term effects and potential relapse rates.

**Table 2:** Detailed summary of study characteristics.

Study	Sample size	Primary indication	Study design	Intervention	Follow-up timeline	Psychiatric measures	Results
Lahtinen, 2020 (46)	N=49	IBS	Randomized Controlled Trial	FMT vs Autologous Placebo	5 follow-up timepoints (4,8,12,26, and 52 weeks)	IBS-QoL, BDI, BAI, and 15D	No significant differences in IBS-QoL, 15D, BDI, or BAI scores. Significant correlation between change in IBS symptoms and change in BDI and 15D when examining treatment groups together.
Huang, 2019 (47)	N=30	Refractory IBS	Open-Label, Single-Arm Trial	FMT	3 follow-up timepoints (1-, 3-, and 6-months)	IBS-QOL, GSRS, HAM-A and HAM-D	The IBS-QoL, and HAMA had a significant improvement in scores at the 1- and 3-month follow-up, but no significant improvement at the 6-month follow-up. The HAMD had a significant improvement in scores at 1-, 3-, and 6-months post-FMT, all with varying significance values.
Johnsen, 2019 (48)	N=90	Moderate to Severe Non-Constipated IBS	Randomized, Double-Blind, Placebo-Controlled, Parallel group, Single Centre	FMT Frozen vs FMT Fresh vs Autologous Placebo	2 follow-up timepoints (6- and 12-months)	IBS-QoL	Patients in the active treatment group achieved clinical improvement in IBS-QoL score from baseline to 6 months compared to placebo group. No significant effect was found at 12 months.
Mizuno, 2017 (49)	N=10	IBS	Single-Centre, Open-Label, Non-Randomized	FMT	2 follow-up timepoints (4- and 12-weeks)	HAM-D, HAM-A	HAM-D scores significantly improved at 4 weeks post-FMT but returned to baseline levels at 12 weeks post-FMT. There were no significant differences for the HAM-A at 4- or 12-weeks post-FMT.
Xie, 2019 (50)	N=1	Male with recurrent GI-related events, alopecia areata, and depression.	Case Study	FMT	1 follow-up timepoint (18-months)	HAM-D	Depressive symptoms on the HAM-D significantly improved.
Cai, 2019 (53)	N=1	Female with decreased appetite, constipation, introversion, and drowsiness.	Case Study	FMT	3 follow-up timepoints (4 days, 2- and 24-weeks)	PHQ-9	PHQ-9 decreased from 21 to 4, indicating a return to normal PHQ-9 levels. Weight returned to normal and constipation symptoms improved.
Lin, 2021 (56)	N=18	IBS	Randomized, single-blind, placebo-controlled	Oral FMT capsules vs Control (placebo capsules)	3 follow-up timepoints (1-week, 1-, 2-, and 3-months)	IBS-QOL, HAM-A, and HAM-D	Patients in FMT group had significant alleviation in anxiety and depression, reflected by continuous decrease of HAM-A and HAM-D over time. Improvement was maintained at 3 months. IBS-QoL also improved in the FMT group at all timepoints. The longer of time after FMT treatment, the greater the effect of improvement (improving more over time).
Huang, 2022 (54)	N=1	Autism Spectrum Disorder	Case Study	FMT	3 follow-up timepoints (1 week, 1-, and 3-months)	HAM-A, HAM-D, and SCL-90	Patient had improvement in HAM-A, HAM-D, and SCL-90 at 1-week and 1-month post-FMT, but this effect was diminished at 3-months post-FMT. Interestingly, digestive issues remained improved compared to pre-FMT.
Ghorbani, 2022 (52)	N=28	Obesity & IR	Phase 2, parallel, double-blind, randomized controlled trial	FMT vs Autologous Placebo	2 follow-up timepoints (1- and 3-months)	HAM-A, MADRS, and QoL	No significant difference between allogenic and autologous groups for QoL. No differences between allogenic and autologous groups for HAM-A or MADRS. However, in the allogenic group, there was a downward trend in anxiety and a significant reduction in depression at 3-months post-FMT.
Wang, 2023 (55)	N=400	Ischemic Stroke	Randomized Study	Cerebral infarction medication + probiotic strains vs ONLY cerebral infarction medication	6 follow-up timepoints (1, 7, 14, 30, 60, and 90 days)	HAM-D	HAM-D scores were significantly lower in the intervention group, than the observation group, across all timepoints.

^15D=Generic Health-Related Quality of Life^ (59)^; BAI=Beck Anxiety Inventory; BDI=Beck Depression Inventory; FMT=Fecal Microbiota Transplantation; GSRS=Gastrointestinal Symptom Rating Scale; HAM-A=Hamilton Anxiety Rating Scale; HAM-D=Hamilton Depression Rating Scale; IBS=Irritable Bowel Syndrome; IBS-QoL=Irritable Bowel Syndrome Quality of Life; PHQ-9=Patient Health Questionnaire; SCL-90=Symptom Checklist-90.^

### 3.3 Primary physical indication

Seven of the ten included studies in this review investigated physical illnesses as a primary indication and psychiatric symptoms as a secondary outcome (46–52). The studies were comprised of the following primary indications: IBS (*n* = 3), refractory IBS (*n* = 1), non-constipated IBS (*n* = 1), obesity and insulin resistance (*n* = 1), and recurrent abdominal pain with non-infectious diarrhea (*n* = 1). The majority of studies involved the use of FMT as the gut-repopulation method from a healthy donor and assessed mood using psychiatric measures. Of the physical indication studies, six studies assessed for depressive symptomology using either the Beck Depression Inventory (BDI), Hamilton Depression Rating Scale (HAM-D), or Montgomery-Asberg Depression Rating Scale (MADRS). The majority of studies reported a sustained improvement in depressive symptoms at least 3-months (*n* = 4) post-FMT (47, 50–52). Interestingly, Huang et al. (2019) reported a sustained improvement in symptoms at 6-months post-FMT and Xie et al. (2019) found the effect lasting up to 18-months post-FMT, in a single case study (47, 50), while others reported no sustained long-term improvement in depressive symptoms (*n* = 2) (46, 49).

Again, of the physical indication studies, five assessed for symptoms of anxiety using either the Beck Anxiety Inventory (BAI) or Hamilton Anxiety Rating Scale (HAM-A) and reflected similar mixed findings. Some studies found a sustained improvement in symptoms of anxiety at 3-months post-FMT (*n* = 2) (47, 51), while the remaining studies did not (46, 49, 52). Some of the physical indication studies (*n* = 3) also utilized the IBS Quality of Life (IBS-QoL), a questionnaire designed to assess overall quality of life for individuals dealing with IBS, with one study using the IBS-QoL as its sole psychiatric measure. Lahtinen et al. (2020) found no significant effect in IBS-QoL scores across any timepoints, while Lin et al. (2021) found a significant improvement at 3-months post-FMT, and Johnsen et al. (2019) found a clinical improvement in scores at 6-months post-FMT (46, 48, 51).

### 3.4 Primary psychiatric indication

The remaining psychiatric-focused studies (*n* = 3) consisted of two case studies (53, 54) and a randomized study (55). As previously mentioned, both case studies have been included due to the novelty of this research area and the lack of available clinical literature. Huang et al. (2022) investigated the use of FMT as treatment for an 18-year-old male patient who was diagnosed with autism spectrum disorder and presented with diarrhea-predominant irritable bowel syndrome (54). The patient’s clinical symptoms were comprised of difficulty communicating with people, feelings of anxiety, impulses of destroying things, and occasional auditory hallucinations and delusions. A psychiatrist conducted the HAM-A, HAM-D, and the Symptom Checklist-90 (SCL-90), a questionnaire used to assess psychological problems, at the following post-FMT follow-ups: 1-week, 1-month, and 3-months post-treatment. Results highlighted that there was a significant improvement in the patient’s mental state at 1-week and 1-month post-treatment, however psychological symptoms reappeared at the 3-month follow-up. Interestingly, while clinical symptoms reappeared at 3-months post-treatment, the patient’s IBS symptoms maintained improvement (54).

On the contrary, Cai et al. (2019) followed the case of a 79-year-old woman diagnosed with depression and presented with loss of appetite, weight loss, constipation, introversion, and drowsiness (53). The patient received FMT via gastroscope and was followed up at 3 timepoints post-treatment: 4 days, 2- and 24-weeks. Using the Patient Health Questionnaire (PHQ-9), a questionnaire used to assess the severity of depression, patient scores improved from 21 at pre-treatment to 4 at 24-weeks post-treatment, indicating a return to average levels (53). Additionally, the patient’s weight also returned to normal and constipation symptoms had improved.

Wang et al. (2023) conducted a randomized study on the effect of combined probiotic and cerebral infarction medications, versus only cerebral infarction medication, on post-stroke depression. Patients were followed up at 6 timepoints post-treatment: 1, 7, 14, 30, 60, and 90 days. Using the HAM-D, scores were significantly lower in the probiotic intervention group, than the observation group, across all timepoints (55).

## 5 Discussion

### 5.1 Main findings

This review presented with mixed findings in terms of evaluating the longitudinal efficacy of microbial therapeutics as treatment for psychiatric disorders. Given that the majority of studies that met inclusion criteria for this review were conducted in populations with a G.I.-related illness and evaluated psychiatric well-being as a secondary outcome measure, no strong conclusions can be made regarding the longitudinal efficacy of microbial treatment use within psychiatry. That being said, the studies available do provide valuable insight into the long-term effects of microbial therapeutics and their associated relapse and/or remission rates. These results also highlight the rarity of studies conducting long-term clinical and biological follow-up assessments, which is absolutely necessary to ascertain if sustained improvements in mood – are related to lasting changes in gut microbiome composition.

Of the nine studies that assessed for depressive symptomology: the majority (*n* = 6) reported a lasting improvement in mood at 3-months post-treatment (47, 50, 52, 53, 55, 56); some (*n* = 2) reported an improvement in symptoms shortly after treatment, but the effect diminished at 3-months post-treatment (49, 54); and one study found no significant reduction in symptoms (46). Interestingly, while both Huang et al. (2022) and Lahtinen et al. (2020) found no sustained improvement in mood, they both reported a lasting reduction in GI-related symptom severity for IBS and refractory-IBS, respectively (46, 54). Huang et al. (2019) reported that the improvement in mood was more significant at 3-months than at 6-months post-treatment, with Johnsen et al. reinforcing this claim by also finding an improvement in mood at 6-months after treatment, but no sustained effect by 12-months (47, 48). On the contrary, both Xie et al. and Cai et al. documented a lasting improvement in mood at 6- and 18-months post-treatment (50, 53). Only two of the six studies that assessed for symptoms of anxiety, reported a sustained improvement in symptoms at 3-months post-treatment (47, 53), however the rest either found short-term improvement or no sustained improvement past 3 months (46, 49, 52, 54).

Based on these mixed findings and lack of consistency between results, it becomes clear that there is a critical need for more randomized, placebo-controlled, studies with a longitudinal component in order to fully assess the long-term potential of microbial therapeutics. Given the recent FDA approval of Rebyota, a fecal microbiota product delivered rectally, and Vowst, a fecal microbiota product delivered orally, there is an increasing sense of urgency for a full characterization of the long-term effects involved with gut microbiome-targeting treatments (57, 58). In efforts to accomplish this, it is pertinent to reach a consensus on the regulation and standardization across the use of microbial therapeutics. A standardization of criteria is not only warranted for creating consistency across CFU dosage ranges, frequency of application, and timing of treatment, but it’s equally as necessary for the selection of healthy donors. Without a clear definition of a ‘healthy’ microbial composition of the gut, it leaves room for discrepancies amongst studies in regard to the criteria used for selecting healthy donors. This ultimately diminishes the consistency and comparability between studies, while also posing as a possible safety risk in terms of the varying degrees of stringency across donors. Studies need to evaluate the long-term effects of these treatments, in diverse populations, with as much transparency as possible, when disclosing the reasoning behind the decisions made regarding healthy donor selection and the procedural methodology.

### 5.2 Limitations

Given the novelty of using microbial therapeutics for psychiatric disorders, there is limited research that exists in this field, especially while considering the long-term efficacy. Though the studies included in this review contributed to a deeper understanding of the long-term effects of microbial therapeutics in the context of psychiatric well-being, some limitations still exist. One significant limitation was the rarity of longitudinal follow-ups in studies with psychiatric disorders as the main indication. All but three studies included in this review assessed non-psychiatric-focused disorders as the primary indication, and not necessarily those exclusively with psychiatric disorders. Hence, any improvement of psychiatric symptoms cannot be entirely attributed to a direct relationship between microbial composition and its influence on mood. Rather, improvements of physical symptoms may result in downstream improvements of psychiatric symptoms. This confounding limitation is evident in any bidirectional relationship, as it becomes indistinguishable if the treatment improved mood directly, or rather, improved mood through the means of improving G.I. symptoms. Many of the studies also had small sample sizes or lack of randomization and placebo-controlled design, making it difficult to generalize results and comment on the long-term efficacy rates.

Another limitation found in this review was the lack of consistency for follow-up duration across all studies. Although our search criteria maintained a minimum follow-up timepoint of 3-months in duration following treatment, it is difficult to compare and contrast multiple studies when they differ in follow-up durations. A study which utilizes a single follow-up timepoint of 18-months post-treatment, even with reported favorable results, cannot be regarded to the same standard as a study that produced favorable results at 6-months post-treatment. With the absence of consistency across data points, it is impossible to suggest that an improvement at 6-months after treatment would also yield an improvement at 18-months post-treatment.

### 5.3 Future directions

Due to the nature of longitudinal studies in clinical settings, confounding variables and extenuating circumstances will always be a factor to consider. It is very possible that any sustained improvements in mood post-treatment, may have also been influenced by environmental stimuli and/or other lifestyle changes between visits. However, the same argument can also be made for many other psychiatric treatment options post-intervention, as environmental stimuli will always be a factor to consider, regardless of treatment choice. Given the uncertainty of whether the gut microbiome reverts back to a pre-treatment state or not, the call for multiple well-documented follow-ups, assessing microbial composition, is needed for establishing the extent and efficacy of long-term stability. In pursuance to solving this challenge, the assessment of microbial diversity at varying frequencies after initial treatment, such as 1-,3-,6-,12-, and 18-months post-FMT, would be highly beneficial. Dependent on those longitudinal findings and varying rates of response or improvement, it is then possible to gauge the need of future booster treatments. It is only through this repetitive process of long-term follow-ups and longitudinal characterization of the gut, that framework and guidelines surrounding the use of microbial therapeutics would be more concrete in its regulation.

## 6 Conclusion

Given the heterogenous nature of psychiatric symptomology and high levels of comorbidity with other physical illnesses, psychiatric disorders have been historically difficult to treat. Though microbial therapeutics have shown promise in restoring and alleviating mood-perturbations, there have been few studies to assess the long-term efficacy of such treatments. Using information available from the studies included in this review, their findings provide some evidence that microbial therapeutics may have the potential to improve measures of psychiatric well-being, with said improvement lasting for 3- to 6-months post-treatment, if not longer. Furthermore, given microbial therapeutics’ unique ability to influence mood either directly or indirectly through the treatment of other physical ailments, using it as an adjunctive therapy with other treatments presents as an option (19).

The results from this review further highlight the need for more long-term follow-up, large-scale, randomized controlled trials, in populations with varying psychiatric disorders. Targeting the gut microbiome as treatment for psychiatric disorders is still a new and quickly growing field, with a limited amount of published research and an even more restricted sub-section assessing longitudinal efficacy. The findings from this review demonstrate intriguing evidence for the potential of microbial therapeutics to produce sustained effects in mood. The extent to which this is maintained and to what degree, is something that only further research can discern.

## Data Availability

The original contributions presented in the study are included in the article/supplementary material. Further inquiries can be directed to the corresponding author/s.
